# Supplementary material for: Spillover of volatility among financial instruments: ASEAN-5 and GCC market study
Source: PLoS One. 2023 Oct 19;18(10):e0292958. doi: 10.1371/journal.pone.0292958 (PMC10586706; doi:10.1371/journal.pone.0292958)
Supplement: Attachment — Submitted filename: Referee_report.docx [file pone.0292958.s001.docx]

Dear Author(s)

I would like to thank the author(s) for your submission and appreciate the opportunity to read and review your manuscript. I enjoyed reading it. From my point of view, it is a very interesting topic. However, I find some recommendations: my comments concern only with clarity, text, and interpretation of results. I would recommend publication, after the issues below are addressed.

**Major clarifications:**

1.	The author(s) are highly advised to mention the implications for research, in the last section. I noted that the authors did not mention how this paper bridges the gap between theory and practice.

2.	Since this paper is solving one of the issues, it is worthy of mentioning how can the research be used in practice? Are there implications consistent with the findings and conclusions of the paper?

3.	The research results need more improvement to present the study findings. Therefore, more effort should be invested in the empirical findings and conclusion sections and tie together the other elements of the paper.

**More comments can raise below:**

1.	The title: a good title should be concise, accurate, and informative, and it should tell the reader exactly what the article is about. Dear authors please rewrite the title.

2.	Abstract: it does not provide an accessible summary of the study.

3.	The Keywords: the keywords does not accurately reflect the content of the study.

Dear author(s) please try to rewrite the title, abstract and keywords to be more optimized for the study purposes.

4.	Dear author(s) to help guide your readers, please end your introduction with the structure of the paper.

5.	Data and Methods section: it needs more clarifications.

6.	Empirical Findings and Conclusion sections: should be better analyzed and developed further, it needs more improvement to present the study findings. Please augment its quality with more depth, rigor and substance.

7.	Please be careful in using the abbreviations and abbreviated words throughout the paper.

8.	Please be careful in using "Punctuating", e.g. (comma, full stop, etc…) throughout the paper.

Finally, based on the above-mentioned comments, I suggest “major revision” of the paper before publication.
